# Anticancer Activity of Polysaccharides Produced from Glycerol and Crude Glycerol by an Endophytic Fungus *Chaetomium* *globosum* CGMCC 6882 on Human Lung Cancer A549 Cells

**DOI:** 10.3390/biom8040171

**Published:** 2018-12-11

**Authors:** Zichao Wang, Peizhang Chen, Ning Tao, Huiru Zhang, Ruifang Li, Xiaobei Zhan, Fuzhuan Wang, Yingben Shen

**Affiliations:** 1 College of Biological Engineering, Henan University of Technology, Zhengzhou 450001, China; chenpeizhang@163.com (P.C.); ningtao@163.com (N.T.); lrf@haut.edu.cn (R.L.); fuzhuanwang@163.com (F.W.); 2 The Key Laboratory of Carbohydrate Chemistry and Biotechnology, Ministry of Education, School of Biotechnology, Jiangnan University, Wuxi 214122, China; xbzhan@yahoo.com; 3 Department of Biochemistry and Molecular Medicine, UC Davis Comprehensive Cancer Center, University of California Davis, Sacramento, CA 95817, USA; ybshen@ucdavis.edu

**Keywords:** *Chaetomium globosum* CGMCC 6882, glycerol, crude glycerol, polysaccharide, anticancer activity

## Abstract

Two polysaccharides were produced by *Chaetomium globosum* CGMCC 6882 from glycerol (GCP-1) and crude glycerol (GCP-2). Chemical characteristics results showed GCP-1 and GCP-2 were similar polysaccharides, but the molecular weights of GCP-1 and GCP-2 were 5.340 × 10^4^ Da and 3.105 × 10^4^ Da, respectively. Viabilities of A549 cells after treatment with GCP-1 and GCP-2 were 49% and 39% compared to the control group. Meanwhile, flow cytometry results indicated that GCP-1 and GCP-2 could induce 17.79% and 24.28% of A549 cells to apoptosis with 200 μg/mL concentration treated for 24 h. RT-PCR results suggested that GCP-1 and GCP-2 could be used as potential and effective apoptosis inducers on A549 cells by increasing *BAX*, *CASPASE-3*, *CASPASE-9*, *TIMP-1*, *TIMP-2* expression and decreasing *BCL-2* expression. This research provided an innovative approach to using a byproduct of biodiesel production (crude glycerol) to produce polysaccharides of potential medicinal benefit.

## 1. Introduction

Fossil fuel exhaustion and global warming have become increasingly serious problems around the world along with the increase of vehicles. There is an extensive and urgent search for alternative and renewable energy resources worldwide. Biodiesel, because of its properties of regeneration and reduction in carbon dioxide emissions, is considered one of the most promising alternatives to fossil fuels [1]. Nowadays, biodiesel is mainly produced by a trans-esterification process from plant or animal oils with methanol as catalyst. However, biodiesel production generates crude glycerol as a byproduct, which accounts for one-tenth of the total biodiesel yield [2]. Biodiesel production grew from 250 million gallons in 2006 to 1.8 billion gallons in 2013 in the United States, and the global output probably reached 37 trillion gallons in 2016 [3]. Although pure glycerol could be widely used in the food, cosmetic and pharmaceutical industries, it is technically difficult and financially costly to purify glycerol from crude glycerol, and the surplus amount of crude glycerol has changed from a byproduct to a burden of the biodiesel industry due to its unsuitability for such applications [4].

*Gynostemma pentaphyllum*, a well-known edible and medicinal plant, has been widely used in beverages, food and traditional herbal medicines [5]. In the past decades, many studies have shown that *G. pentaphyllum* has a variety of biological activities, such as anticancer, anti-tumor, anti-gastric ulcer, anti-inflammation, cardio-protection and strengthening the immune system [6]. Although the *G. pentaphyllum* herb contains the active ingredients saponin, amino acids, flavonoids, essential oils and polysaccharides, polysaccharides may have greater bioavailability than others because they are water soluble and can be easily soaked out into a tea infusion [7,8]. Many evidences have shown that the polysaccharide components obtained from *G. pentaphyllum* herbs exhibited significant bioactivities, including antioxidant, anti-cancer, anti-tumor, anti-aging, immunomodulatory and anti-exercise fatigue effects [5].

Endophytic fungi are a group of microorganisms that live in plant tissues and organs for all or part of their life cycle, but do not cause any apparent harms or pathogenic infections to their host plants [9,10]. Meanwhile, many researchers have already reported that some of these endophytic fungi could synthesize the same or similar bioactive secondary metabolites to their host plants [11,12]. The properties of endophytic fungi provide a new approach for the production of bioactive compounds through industrial fermentation. This study investigated the anticancer activity of polysaccharides produced by an endophytic fungus *Chaetomium globosum* CGMCC 6882, which was isolated from *G. pentaphyllum* using glycerol and crude glycerol. At first, the polysaccharides were extracted and purified from the fermentation liquid. Secondly, the chemical characteristics of the polysaccharides, such as monosaccharide composition, molecular weight, functional groups and chemical bonds, were analyzed by HPAEC (high performance anion exchange chromatography), HPSEC (high performance size exclusion chromatography), FT-IR (Fourier transform infrared spectroscopy) and NMR (nuclear magnetic resonance spectroscopy). In the end, the effects of GCP-1 and GCP-2 on A549 cell viability, apoptosis, and apoptosis- and metastasis-related genes were studied.

## 2. Materials and Methods

### 2.1. Microorganism and Culture Medium

#### 2.1.1. Microorganism

*Chaetomium globosum* CGMCC 6882, which was isolated from *G. pentaphyllum* root and stored at the China General Microbiological Culture Collection Center (Beijing, China), was employed in this work. It was stored on potato dextrose agar (PDA) plates at 4 °C and transferred monthly, and fungal strain spores obtained from the agar surface were maintained in 30% glycerol at −80 °C for long-term storage.

#### 2.1.2. Preparation of Seed Medium

Spores of *C. globosum* CGMCC 6882 were collected by scratching the PDA plates with a disposable spreader and washing with sterile distilled water. Then, a hemacytometer (Neubauer, Marienfeld, Germany) was used to count the number of spores under a phase contrast microscope (Zeiss, Axio Imager 2, Jena, Germany). Then, the spore suspension was diluted to a concentration of 1 × 10^7^ spores/mL with distilled water; the inoculation volume was 1% (V/V).

#### 2.1.3. Batch Fermentation

The fermentation medium used for *C. globosum* CGMCC 6882 consisted of 40 g/L glycerol (or crude glycerol), 1 g/L peptone, 1 g/L yeast extract, 1 g/L beef extract, 1.5 g/L MgSO_4_·7H_2_O, 1 g/L KH_2_PO_4_, and 1 g/L K_2_HPO_4_. The fermentation conditions were: temperature, 28 °C, and cultivation time, 7 d. The pH was maintained at 7.0 ± 0.02 with 1 mol/L NaOH and 1 mol/L HCl; 7.0 L fermentor (BioFlo 115, New Brunswick, USA) with 3.5 L working volume; agitation and aeration were set to 100 rpm and 0.8 vvm during the fermentation process, respectively. The component of the crude glycerol used in this work was the same as in our previous study [13]. All batch cultures were performed in three replications.

### 2.2. Characterization of GCPs Produced from Glycerol and Crude Glycerol

At the end of fermentation, the culture broth was filtered and centrifuged at 12,000× *g* for 30 min to remove mycelium and cells. Then, the broth was concentrated to 1/10 of the original volume with rotary evaporation at 60 °C and 0.1 MPa vacuum. After that, the concentrated broth was deproteinized by adding three volumes of Sevag solution (chloroform:butyl alcohol = 4:1), and three volumes of cold 95% alcohol was added to the deproteinized supernatant and kept at 4 °C overnight to precipitate the GCP. The precipitated GCP was re-dissolved in distilled water and de-pigmented with AB-8 macroporos resin, and then the GCP solution was filtered through a 0.22 μm filter and applied to a Sepharose CL-6B column (Sigma-Aldric, Missouri, USA) (2.5 × 60 cm) for further purification, eluting with 0.1 mol/L NaCl solution at a flow rate of 0.6 mL/min. In the end, the fraction was collected and freeze dried to study the molecular characteristics and anti-cancer activities of GCPs.

High performance anion exchange chromatography (HPAEC) was used to analyze the monosaccharide composition of GCPs. GCPs were dissolved in 2 mol/L trifluoroacetic acid (TFA) and hydrolyzed at 120 °C for 2 h, then the hydrolysate was washed three times with methanol and evaporated to dryness to remove TFA. The hydrolyzed material was transferred to a 25 mL volumetric flask, diluted to 25 mL by deionized water and subjected to a Dionex ICS5000 system (Dionex, Sunnyvale, California, USA) equipped with CarboPac PA20 column (ID 3 mm × 150 mm). The mobile phase was deionized water (A) 0.25 mol/L NaOH (B) 1 mol/L NaAc (C) and eluted as follows (A%, B%, C%): 0 min: 99.2, 0.8, 0; 30 min: 99.2, 0.8, 0; 40 min: 79.2, 0.8, 20; 40.1 min: 20, 80, 0; 60 min: 99.2, 0.8, 0. The flow rate was 0.45 mL/min and the injection volume was 25 µL. The column temperature was 30 °C and detected by a pulsed ampere detector, Au electrode, and Ag/AgCl reference electrode. The standard monosaccharides used in this work were fucose, rhamnose, arabinose, glucosamine, galactose, glucose, xylose, mannose, fructose, galacturonic acid and glucuronic acid.

GCPs were dissolved in distilled water to a concentration of 2 mg/mL and analyzed by high performance size exclusion chromatography (HPSEC) for their molecular weights. The system consisted of Waters 2695 HPLC system equipped with multiple detectors: a refractive index detector (RI) and a UV detector for concentration determination, a multiple angle laser light scattering detector (Wyatt Technology, Sant Barbara, USA) for direct molecular weight determination and a differential pressure viscometer (DP) for viscosity determination. The columns were TSK PWXL 6000 and 3000 gel filtration columns (Sigma-Aldrich, Pennsylvania, USA), which were eluted with PB buffer (0.15 mol/L NaNO_3_ and 0.05 mol/L NaH_2_PO_4_, pH = 7) at the flow rate of 0.5 mL/min. The calibration of the laser photometer was performed with ultrapure toluene. The normalization was conducted with a bovine serum albumin globular protein (Mw = 66.7 kDa, Rg = 2.9 nm). A value of 0.146 mL/g was used as the refractive index increment (dn/dc) for molecular weight calculation. Astra software (Version 6.1.1) (Wyatt Technology, Sant Barbara, USA) was utilized for data acquisition and analysis. The column temperature and RI detector temperature were maintained at 35 °C.

A nexus 470 FT-IR spectrophotometer (Nicolet, NY, USA) was used to record the IR spectrum of GCP with KBr between 400 and 4000 cm^−1^, and a Bruker Avance 400 MHz spectrometer (Bruker Inc., Germany) was used to record the ^1^H NMR spectra of GCPs at 30 °C. GCPs were dissolved in D_2_O in 5 mm OD NMR tubes, the chemical shifts for ^1^H NMR spectra were recorded in parts per million from tetramethylsilane.

### 2.3. MTT Reduction Assay and Lactate Dehydrogenase (LDH) Activity Assay

The effect of GCP-1 and GCP-2 on A549 cells viability was detected by using MTT dye reduction assay and lactate dehydrogenase (LDH) activity assay. A549 cells were placed in 96-well plates with a density of 1 × 10^5^ cells/mL and cultured at 37 °C with 5% CO_2_ for the cells to attach. After 24 h incubation, A549 cells were treated with various concentrations (50, 100, 200 μg/mL) of GCP-1 and GCP-2 and incubated at 37 °C with 5% CO_2_ for another 24 h. Under the same conditions, distilled water was used as control. After various treatments, 10 μL MTT (5 mg/mL) was added to each well and incubated for another 4 h at 37 °C. Then, the medium was aspirated and 200 μL DMSO was added to each well to dissolve the purple formazan dye. The absorbance at 490 nm was detected by a microplate reader (Tecan, GENios ELIASA Co., Salzburg A-5082, Austria), and the cell viability was expressed as a percentage compared to the control group. At the same time, the amount of LDH released into the culture medium was detected according the manufacturer’s protocol. The colorimetric absorbance at 490 nm was immediately detected by the microplate reader and the LDH activity was expressed as a percentage of released LDH to the control group.

### 2.4. Apoptosis Analysis by Annexin V-FITC/PI

Briefly, A549 cells were seeded in 24-well plates to a density of 1×10^5^ cells/mL and exposed to 200 μg/mL GCP-1 (or GCP-2) for 24 h at 37 °C with 5% CO_2_. PBS was used as control. At the end of the treatment, cells were harvested and washed twice in ice-cold PBS, then re-suspended in 250 μL binding buffer, after that, the cell suspension was immediately incubated with 5 μL Annexin V-FITC (Bio Legend Inc., San Diego, CA, USA) and 10 μL propidium iodide at room temperature for 15 min in darkness. The distribution of cell apoptosis was detected by a flow cytometer (BD FACScan Calibur, New Jersey, USA)

### 2.5. Real-Time Reverse Transcription Polymerase Chain Reaction (RT-PCR) Analysis

A549 cells were seeded in 24-well plates and incubated at 37 °C in an atmosphere of 5% CO_2_ to a concentration of 1 × 10^5^ cells/mL. After that, the culture medium in the 24-well plate was discarded and the A549 cells were washed twice with 37 °C PBS (phosphate buffer saline). Then, the 24-well plate was added with 1 mL 200 μg/mL GCP-1 (or GCP-2) and cultured for 8 h, PBS was used as control. Finally, the culture medium was discarded and A549 cells were washed twice with ice-cold PBS for cell collection. The collected cells were lysed with cell lysis buffer and the total RNA was extracted according to the manufacturer’s instructions with a Total RNA Isolation Kit (Sangon, Shanghai, China). β-actin was used as the internal control and RT-PCR was conducted in triplicate for each sample. A total of 40 cycles of 95 °C 10 s, 60 °C 10 s, and 72 °C 30 s were carried out for PCR amplification. The PCR product was analyzed using 2.0% (*w*/*v*) agarose gel electrophoresis and then stained with ethidium bromide. The band intensity was analyzed by using Bio-Imaging System with the Quantity One 1-D analysis software (Bio-Rad, CA, USA). The PCR primers used in this work were as follows: 5′-CCTTCCTTCCTGGGCATGG-3′ (Forward) and 5′-AGTGATCTCCTTCTGCATCC-3′ (Reverse) for β-actin gene; 5′-GGAGCTGCAGAGGATGATTG-3′ (Forward) and 5′-GGCCTTGAGCACCAGTTT-3′ (Reverse) for *BAX* gene; 5′-GTGGATGACTGAGTACCTGAAC-3′ (Forward) and 5′-GAGACAGCCAGGAGAAATCAA-3′ (Reverse) for *BCL-2* gene; 5′-GAGCCATGGTGAAGAAGGAATA-3′ (Forward) and 5′-TCAATGCCACAGTCCAGTTC-3′ (Reverse) for *CASPASE-3* gene; 5′-CGACCTGACTGCCAAGAAA-3′ (Forward) and 5′-CATCCATCTGTGCCGTAGAC-3′ (Reverse) for *CASPASE-9* gene; 5′-GCGTTATGAGATCAAGATGACCA-3′ (Forward) and 5′-AACTCCTCGCTGCGGTT-3′ (Reverse) for *TIMP-1* gene; 5′-GCTGCGAGTGCAAGATCA-3′ (Forward) and 5′-CTCTTGATGCAGGCGAAGAA-3′ (Reverse) for *TIMP-2* gene.

## 3. Results and Discussion

### 3.1. Isolation and Purification of Polysaccharides

**Figure 1:**
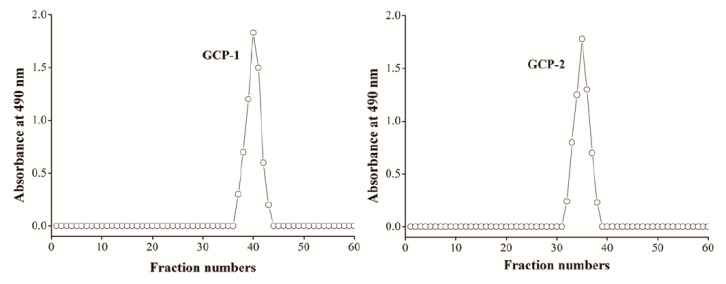
Elution profiles of GCP-1 and GCP-2 in the Sepharose CL-6B column chromatography.

After the fermentation liquid was collected, filtered, and centrifuged at 12,000× *g* for 30 min to remove mycelium and cells, it was de-proteinized by adding three volumes Sevag solution, precipitated by adding three volumes of cold 95% alcohol and kept at 4 °C overnight, washed with 75% cold alcohol, de-pigmented with AB-8 macroporos resin, dialyzed against distilled water and further purified by Sepharose CL-6B column (Sigma-Aldric, Missouri, USA) (2.5 × 60 cm) with 0.1 mol/L NaCl solution at a flow rate of 0.6 mL/min. Two purified and homogeneous polysaccharides (GCP-1 and GCP-2) were obtained with characteristic absorption at 490 nm (Figure 1). Meanwhile, no protein or nucleic acid were detected at 260 or 280 nm by UV spectrum.

### 3.2. Characteristics of GCPs Produced from Glycerol and Crude Glycerol

#### 3.2.1. Monosaccharide Composition and Molecular Weight of GCP-1 and GCP-2

The molecular properties of microbial exopolysaccharides, including monosaccharide composition and molecular weight, are most importantly affected by microorganism species and fermentation conditions used in the production [14]. Table 1 shows that the monosaccharide composition of the GCP-1 produced from glycerol by *C. globosum* CGMCC 6882 was galactose, glucose, mannose and glucuronic acid in a molar ratio of 5.95:58.75:5.65:0.76. Meanwhile, the monosaccharide composition of GCP-2 produced from crude glycerol was also galactose, glucose, mannose and glucuronic acid, but in a molar ratio of 8.16:43.77:5.84:0.43. Moreover, the polydispersities (M_w_/M_n_) of GCP-1 and GCP-2 were 1.012 and 1.076, respectively, indicating their homogeneity. However, the weight-average molecular weight (M_w_) of GCP-2 produced from crude glycerol was 3.105 × 10^4^ Da, which was almost half that of the GCP-1 (5.340 × 10^4^ Da) produced from glycerol.

#### 3.2.2. FT-IR Spectroscopy of GCP-1 and GCP-2

**Figure 2:**
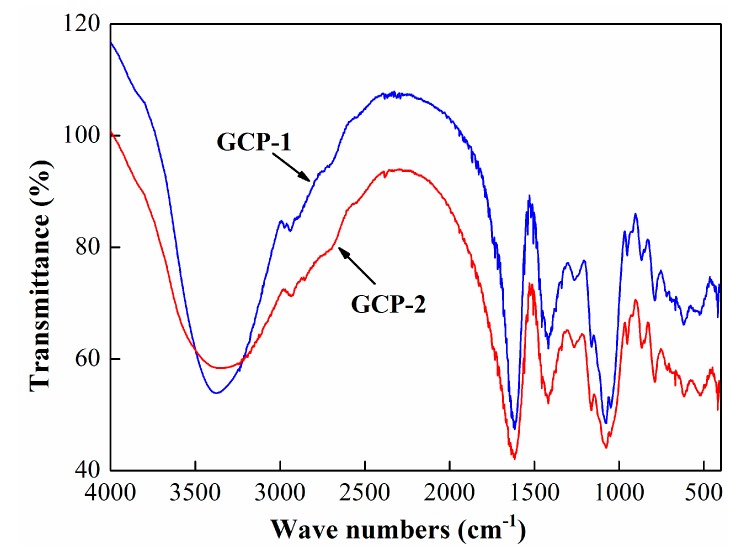
FT-IR spectra of GCP-1 and GCP-2 over the range of 400–4000 cm^−1^.

The FT-IR spectra of GCP-1 and GCP-2 are presented in Figure 2 and Table 2. The strong absorption band at around 3400 cm^−1^ might be assigned to hydroxyl stretching vibration. The weak characteristic absorption peaks of GCP-1 and GCP-2 that appeared at around 2940 cm^−1^ might be attributed to the stretching vibration of O-H and asymmetric vibration of C-H [15]. The absorption peaks at around 1625 cm^−1^ and 1430 cm^−1^ were related to the vibration of the carboxyl group in GCP-1 and GCP-2 [16]. The absorption bands within the range of 1000 cm^−1^ and 1150 cm^−1^ were attributed to the stretching vibrations of C-O and C-C [17]. All these were representative absorption bands of polysaccharides.

#### 3.2.3. NMR Analysis of GCP-1 and GCP-2

**Figure 3:**
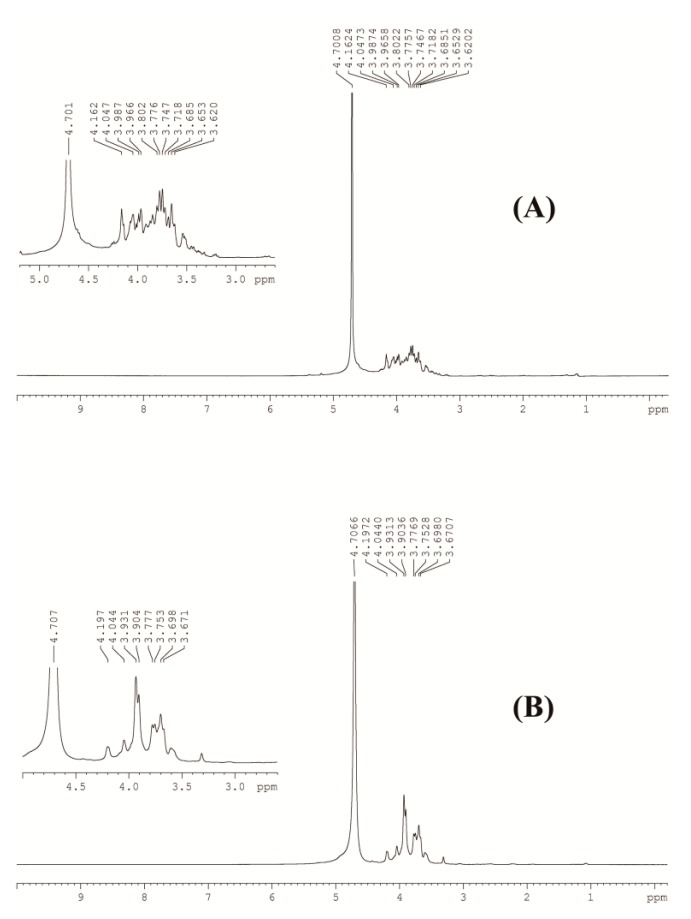
^1^H NMR spectra of GCP-1 (**A**) and GCP-2 (**B**) in D_2_O.

NMR analysis was used to further characterize the structural characteristics of GCP-1 and GCP-2, and the results are shown in Figure 3 and Table 3. The ^1^H signal at around 4.7 ppm could be assigned to the standard material of D_2_O used in this work. The ^1^H signals located within 3.6 ppm and 4.0 ppm corresponded to the H in the hydroxyl group of GCP-1 and GCP-2 [14]. The signal that appeared at around 4.2 ppm was probably due to to the methylene in −COOC_2_H_5_ [18]. Taking the results of the monosaccharide composition and FT-IR spectra into account, GCP-1 and GCP-2 were similar polysaccharides except for the difference in molecular weight.

### 3.3. MTT and LDH Assays

**Figure 4:**
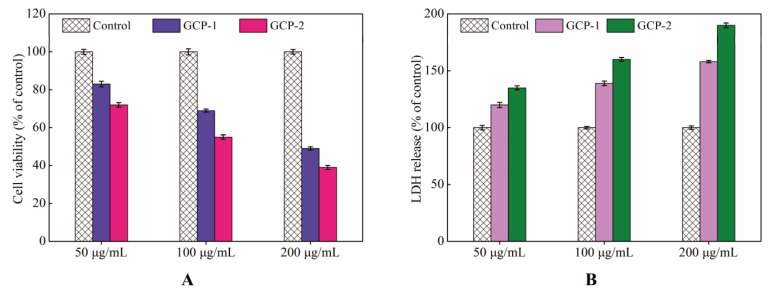
Effects of GCP-1 and GCP-2 on the viability (**A**) and the level of lactate dehydrogenase (**B**) in lung cancer A549 cell lines.

Lung cancer is considered the most common cause of cancer-related death in the world and many studies indicate that polysaccharides possess anti-cancer activity [19,20]. To detect the inhibition effects of GCP-1 and GCP-2 on A549 cells, the MTT method was used to determine the cell viability after being treated with various concentrations (50, 100, 200 μg/mL) of GCP-1 and GCP-2 for 24 h. Figure 4A demonstrates that the proliferation inhibitions of GCP-1 and GCP-2 on A549 cells were in a concentration-dependent manner. After treatment with 50 μg/mL GCP-1 and GCP-2 for 24 h, the cell viabilities of the A549 cells were 83% and 72% compared to the control group. However, when the polysaccharide concentration increased to 200 μg/mL, the cell viabilities decreased to 49% and 39%, respectively. At the same time, the lactate dehydrogenase (LDH) assay, which measures the release of the cellular enzyme into the culture medium to evaluate the loss of cell membrane integrity, could be used as a cell viability indicator [21].

As shown in Figure 4B, the LDH release assessment obtained similar results to the MTT assay in a concentration-dependent manner. Upon exposure to 50 μg/mL of GCP-1 (or GCP-2) for 24 h, LDHs released from A549 cells were 1.2 and 1.4 times greater compared to the control group. This increased to 1.6 and 1.9 when the concentration of GCP-1 and GCP-2 increased to 200 μg/mL. These results suggest that GCP-1 and GCP-2 had potent growth inhibition effects on A549 cells in vitro. Moreover, Figure 4 shows that the inhibition effect of GCP-2 on A549 cells was higher than that of GCP-1; this might be due to the low molecular weight of GCP-2. Many studies have reported that the activity of a polysaccharide is strongly associated with its molecular weight, and polysaccharides with high molecular weight struggle to penetrate the cell membrane and exert a pharmacological effect [22,23]. In previous work, we also found that the entrance of a *G. lucidum* polysaccharide (GLP) with 108 kDa into a cell was through the pathway of macropinocytosis [24].

### 3.4. Cell Apoptosis Analysis

**Figure 5:**
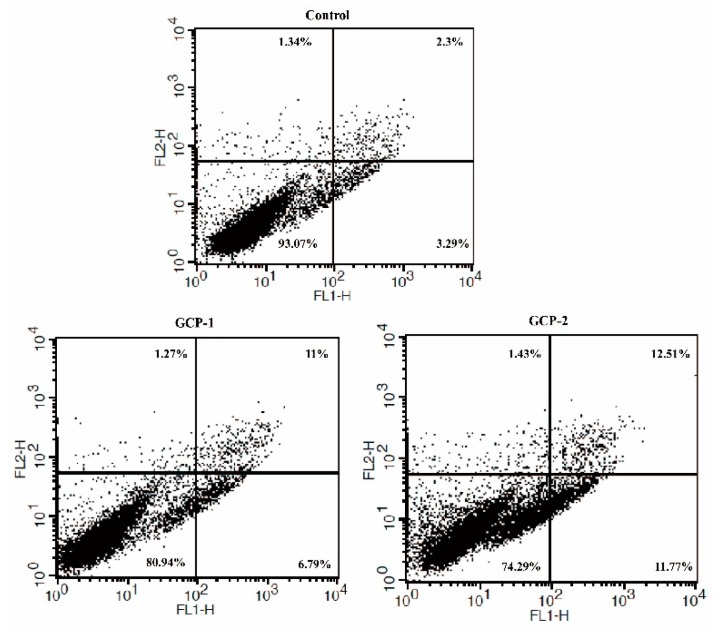
Flow cytometric analysis of apoptosis after treatment with GCP-1 and GCP-2 in human lung carcinoma A549 cell lines using Annexin V-FITC/PI.

In order to investigate whether the induction of cell death by GCP-1 and GCP-2 was related to apoptosis, flow cytometric analysis was used on A549 cells stained simultaneously with Annexin V-FITV/PI. As seen in Figure 5, the incubation of A549 cells with 200 μg/mL GCP-1 (or GCP-2) for 24 h caused cell apoptosis, and the proportion of apoptotic cells in the control, GCP-1 group and GCP-2 group were 5.59%, 17.79% and 24.28%, respectively. Although the effects of apoptosis induced by GCP-1 and GCP-2 were not significant, these results suggested that the anti-proliferative effects of A549 cells by GCP-1 and GCP-2 might partly contribute to apoptosis.

### 3.5. Analysis of Gene Expression by RT-PCR

**Figure 6:**
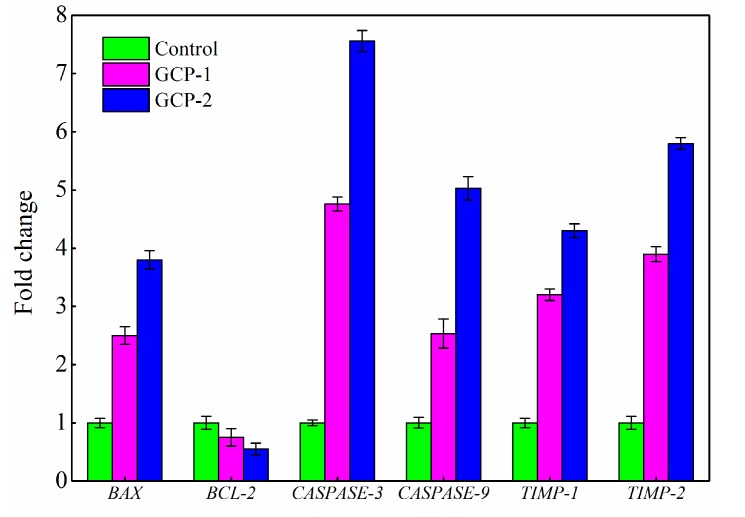
Relative transcription level of apoptosis and metastasis genes before and after treatment with 200 μg/mL GCP-1 and GCP-2.

During cancer treatment, apoptosis plays a very important role due to its programmed cell death. *BAX* is a pro-apoptotic gene and *BCL-2* is an anti-apoptotic gene in *BCL-2* family genes, and the ratio of *BAX* to *BCL-2* plays a key role in the induction of apoptosis in the mitochondrial-mediated apoptosis pathway [25,26]. CASPASE-3 and CASPASE-9 proteins, known as cysteine proteases, are two key proteins in the mitochondrial-mediated apoptosis pathway, and the activation of *CASPASE-3* and *CASPASE-9* are considered gene hallmarks of apoptosis [27]. According to the RT-PCR results of in Figure 6, the expressions of the *BAX*, *CASPASE-3* and *CASPASE-9* genes were 2.48, 4.76 and 2.53 times greater in the GCP-1 treated group compared with the control group, and these values increased to 3.82, 7.56 and 5.03 when the A549 cells were treated with GCP-2. However, the expression of *BCL-2* was determined as 0.75 and 0.53 times in the GCP-1 and GCP-2 treated groups compared to the control group cells.

Metastasis is an important cellular marker of cancer progression and is responsible for approximately 90% of cancer-related deaths [26]. Matrix metalloproteinases, which degrade the connective tissues of the extracellular matrix and promote the invasion of cancer cells, have been associated with metastasis. TIMP-1 and TIMP-2 are tissue inhibitors of metalloproteinases, and the increased expression of these two genes could decrease the cancer cell invasions [28]. In this work, the expressions of *TIMP-1* and *TIMP-2* in the GCP-2 treated group were 4.36 and 5.85 times greater compared with control, and the expressions of *TIMP-1* and *TIMP-2* were 3.27 and 3.98 times greater compared to control in GCP-1 treated group (Figure 6). The above results indicated that GCP-1 and GCP-2 could be apoptosis inducers mediated by mitochondria on A549 cells through the increase of *BAX*, *CASPASE-3*, *CASPASE-9*, *TIMP-1*, *TIMP-2* expression and decrease of *BCL-2* expression.

## 4. Conclusions

The production of active compounds from fermentation could maintain and sustain the development of traditional Chinese medicine resources. An endophytic fungus *C. globosum* CGMCC 6882 can use glycerol and crude glycerol to produce polysaccharides (GCP-1 and GCP-2). A cell viability assay showed that GCP-1 and GCP-2 could inhibit A549 cell growth and flow cytometry analysis suggested that the anti-proliferative effect of A549 cells by GCP-1 and GCP-2 was partly due to the induction of apoptosis. The RT-PCR results indicated that cell apoptosis was induced by the increased expression of pro-apoptosis and anti-invasion-related genes, and the decreased expression of anti-apoptosis-related genes. However, the reason for the higher anti-cancer activity of GCP-2 compared to GCP-1 was not clear, and these two polysaccharides will be further characterized to investigate the structure and activity relationship of GCP-2 and GCP-1 in future work.

## Figures and Tables

**Table 1:** Characteristics of GCP-1 and GCP-2 produced from glycerol and crude glycerol by *C. globosum* CGMCC 6882.

Items	GCP-1	GCP-2
Galactose (umol/L)	5.95	8.16
Glucose (umol/L)	58.75	43.77
Mannose (umol/L)	5.65	5.84
Glucuronic acid (umol/L)	0.76	0.43
Weight-average molecular weight (Mw, Da)	5.340 × 10^4^	3.105 × 10^4^
Number-average molecular weight (Mn, Da)	5.276 × 10^4^	2.885 × 10^4^
Polydispersity (Mw/Mn)	1.012	1.076

**Table 2:** FT-IR spectra assignment of polysaccharide functional groups.

Absorption Band (cm^−1^)	Functional Groups
3400	hydroxyl stretching vibration
2940	O-H stretching vibration and C-H asymmetric vibration
1625	carboxyl vibration
1430	carboxyl vibration
1150	C-C stretching vibration
1000	C-O stretching vibration

**Table 3:** ^1^H NMR spectra assignment of polysaccharide chemical bonds.

Absorption Signal (ppm)	Chemical Bonds
4.7	H in D_2_O
4.2	methylene in –COOC_2_H_5_
4.0	H in hydroxyl group
3.6	H in hydroxyl group
